# Immunology of Normal Pregnancy and Preeclampsia and the Role of Placental Non-Classical HLA and Decidual NK Cells

**DOI:** 10.3390/ijms27177672

**Published:** 2026-08-27

**Authors:** Rinat Hackmon, Dan E. Geraghty, Caroline E. Dunk

**Affiliations:** 1 Obstetrics and Gynecology/Maternal Fetal Medicine (ObGyn/MFM), Oregon Health and Science University (OHSU), Portland, OR 97239, USA; 2 Fred Hutchinson Cancer Research Institute, Seattle, WA 98109, USA; geraghty@fhcrc.org; 3 Consultant in Cellular and Immune-Mediated Maternal-Fetal Communication, Toronto, ON M4C 3B6, Canada; cedunk@gmail.com

**Keywords:** non-classical human leukocyte antigen, human leukocyte antigen-E, human leukocyte antigen-G, human leukocyte antigen-E/G complex, soluble-G, decidual natural killer cell, immunology, pregnancy, preeclampsia, biomarker

## Abstract

Pregnancy is considered a unique immunological process in which contact between the maternal innate immune system and the placental allograft results in maternal tolerance to paternally derived antigens. A growing body of research indicates that interactions between non-classical placental Human Leukocyte Antigen (NCHLA) and receptors on decidual Natural Killer (dNK) cells from early implantation are crucial to this process. Preeclampsia (PE), one of the leading causes of maternal and fetal morbidity and mortality, is also considered an autoimmune process. Currently, there is no treatment for PE except delivery. Most adverse outcomes derive from delayed diagnosis, while newer preventative therapies significantly improve outcomes. An early predictor of PE would enable universal screening, detect and treat high-risk populations earlier, and improve outcomes. Recently, we discovered that high placental HLA-E and G expression occurs during early normal pregnancies, and that placental NCHLA expression differs in PE. Others described the HLA-E/G complex, a potent immunosuppressor of dNK. Interestingly, dNK cells were found to have memory-like properties in binding to HLA-G and HLA-E. The theory proposed is that the HLA-G complex plays a major role in the etiology of PE. In this narrative review, we examine the relevant literature and present our recent findings and those of others that suggest a screening model for PE.

## 1. Introduction

Human pregnancy is one of the most exciting examples of immune tolerance in mammalian biology, in which direct contact between the maternal innate immune system and the placental allograft results in maternal tolerance to paternally derived antigens and supports the development of the placenta and fetus throughout pregnancy [1].

Preeclampsia (PE) is a multisystem disease unique to human pregnancy that complicates 2–7% of all pregnancies and is a leading cause of maternal and fetal morbidity and mortality worldwide [2,3]. In the U.S., the annual number of maternal deaths is ~1205, along with 10,500 fetal deaths, a third of which are associated with PE [4]. Currently, there is no treatment for PE except for delivery due to concerns of maternal and fetal morbidity, resulting in PE being the leading cause of iatrogenic preterm births (PTB) and the most common cause of fetal mortality and morbidity [5].

PE is considered a “great obstetrical syndrome” of unknown etiology; however, within that ambiguity, growing evidence supports the concept of PE as an immune-mediated inflammatory response originating from abnormal placentation early in pregnancy. From this immunological perspective, many researchers consider placentation in the first stage of pregnancy to be graft acceptance, whereas parturition, toward the last stage of pregnancy, is graft rejection. This latter, graft-rejection theory is primarily supported by the pioneering work of Sacks et al. [6] which indicates a maternal systemic inflammatory response in normal third trimester pregnancies. Similar immune-mediated inflammatory reactions have been described in PE, as well as the physiological changes, including edema and an increase in maternal blood pressure, that occur during normal pregnancy and parturition. The above led to the concept that PE may be considered part of the spectrum of normal pregnancy, albeit at an earlier stage [6].

Implantation and placentation during the first trimester of pregnancy 1–13 weeks of gestation (wga) are the foundation of normal fetal development and a healthy pregnancy [7]. The initial development of the placenta involves crosstalk among fetal and maternal cell types, including trophoblasts and decidual cells. Recent findings reveal that maternal natural decidual NK (dNK) cells and placental non-classical Human Leukocyte Antigen (NCHLA) play a principal role in this interplay at the maternal–fetal interface [8].

In this review, we will aim to illuminate the critical role of placental non-classical HLA expression in the proper placental maturation and the reciprocal crosstalk of the placental–maternal–fetal interface since the early stage of implantation. We will first provide an overview of the clinical characteristics of pregnancies and PE. Then we will further review current perspectives on PE prediction and prevention with therapeutic agents. Next, we will discuss the essential role of normal placentation in pregnancy, as well as the immunological aspects of the maternal–fetal interface and their interrelationships. We will also review the latest findings on dNK cells and their receptor-mediated interactions with placental HLA, as well as the expression of placental non-classical HLA in normal pregnancies. Finally, we will discuss recent evidence of dysregulation of non-classical HLA expression in preeclamptic pregnancies, its presumed role in the etiology of PE, and its potential role as an early biomarker of PE.

## 2. Theorem

We hypothesize that dysregulation of placental decidual crosstalk mediated by non-classical placental HLA proteins and interactions with maternal immune cells in the decidua early in pregnancy may contribute to excessive inflammation and maternal immunologic maladaptation that result in PE. The primary objective in this review is to provide supporting evidence for this hypothesis.

## 3. Methods

The narrative review integrates and synthesizes findings from selected articles, providing a comprehensive overview of the role of nonclassical HLA in pregnancy and offering valuable insights into the etiology of immunological processes during pregnancy, as well as potential diagnostic and therapeutic applications.

The search strategy involved conducting a computer-based online search of the Cochrane Library, Embase, Medline, and PubMed to retrieve articles published between January 1990 and January 2025.

The search criteria included the following words: Pregnancy, Placenta, Preeclampsia PE, Decidua, non-classical human leukocyte Antigen (HLA), HLA-G, HLA-E, HLA-C, and HLA-F decidual natural Killer (dNK)cells.

## 4. The Clinical Aspects of PE

PE is a syndrome characterized by maternal new-onset hypertension (HTN) and organ-related effects beyond 20 wga [2,3]. Indeed, the clinical diagnosis of PE is usually established after 20 wga. However, substantial evidence indicates that abnormal implantation and placentation in the early stages of pregnancy are among the essential etiological factors in the development of PE, attributed to defective invasion of the placenta into the uterine spiral arteries.

From a maternal cardiovascular perspective, it is thought that in PE, there is also reduced maternal cardiovascular adaptation and cardiac failure to remodel and accommodate the increased maternal blood volume required to supply the placenta and the developing fetus. Indeed, in preeclamptic women, decreased end-diastolic volume, stroke volume, and cardiac output increase vascular resistance, raising mean arterial pressure and resulting in HTN.

The likelihood of being affected by PE is substantially increased in first pregnancies, multifetal gestations, a prior history of PE, preexisting maternal chronic hypertension (cHTN), diabetes mellitus, autoimmune diseases, advanced maternal age, a new male partner, elevated maternal BMI, etc. [9]. In general, first-time pregnancies in women who have never been pregnant (primigravidae) are considered to have less favorable outcomes than those who have had multiple pregnancies (multigravidae). This interesting finding is based on numerous studies employing various approaches, including population-based epidemiological research, benchmark studies that incorporate histological analysis of the maternal–fetal interface, and molecular-based examinations of epigenetic memory [10].

PE is clinically classified by time of onset and severity. Severe PE is defined when systolic blood pressure (BP) is ≥160 mmHg, and/or diastolic BP is ≥110 mmHg, or any elevated BP with maternal target-organ effects indicating kidney and liver malfunction [11]. Early-onset PE is traditionally defined as severe PE diagnosed <34 wga. Late-onset PE occurs at ≥37 wga. Several groups studied a subgroup of PE with delivery <37 wga, defined as Preterm PE [12,13].

If left untreated, maternal pulmonary edema, eclampsia, brain injury, renal and hepatic failure, disseminated vasculopathy, and maternal death can occur. In multiple instances, placental perfusion is affected by PE, attenuating fetal growth, resulting in fetal growth restriction (FGR) with increased risk for intra-uterine fetal distress that, in extreme cases, may lead to fetal demise [14,15,16]. The earlier PE occurs, and when severe, the more critical the disease is and its adverse outcome, often requiring induced delivery at early fetal prematurity or even before fetal viability, i.e., at 21–22 wga [14]. Notably, most of the significant adverse outcomes stem from delayed diagnosis and delayed access to care, both of which are preventable [17]. Furthermore, the long-term outcome of women who have experienced severe PE in pregnancy is about double the risk of lifetime cardiovascular diseases, including cardiovascular death, coronary artery disease, heart failure, and stroke, in comparison to women with normal pregnancies, and after adjusting for potential confounders. These risks are evident during the first 1–3 years of follow-up and remain significant for more than 10 years [18,19].

Based upon the above, two distinct phenotypes of PE have recently been proposed: Type I presents earlier (<34 wga) and is characterized by placental dysfunction caused by shallow trophoblast invasion, inadequate spiral artery conversion evidenced by abnormal uterine artery Doppler, profound syncytiotrophoblast stress, manifested by elevated soluble fms-like of tyrosine kinase-1 (sFlt-1) levels and reduced placental growth factors levels, high maternal peripheral vascular resistance with low maternal cardiac output, and often with an FGR. Type II is typically diagnosed in the later stages of pregnancy and entails an evolving maternal cardiac response to the demands of pregnancy, with a moderately dysfunctional placenta and inadequate blood supply. In severe cases, the adverse outcome may resemble that of Type I [20,21].

## 5. Current Perspectives on Predicting PE

An early predictor or biomarker of PE would provide a universal screening tool, enable earlier detection and prevention in high-risk populations, and enable testing of novel prevention and treatment strategies to alter the clinical course of PE and significantly improve health outcomes [11,22]. Indeed, the US Preventive Services Task Force emphasized the importance of “identifying women at risk as this remains a ‘holy grail’ of modern obstetrics and a task worth striving for” [23]. Despite ongoing work and extensive research, we still cannot accurately predict PE early in pregnancy, when prevention remains feasible. Below is a summary of the most significant findings in the search for early PE biomarkers and modalities used in the field:

### 5.1. Angiogenic Growth Factors

In a healthy pregnancy, the angiogenic placental growth factor (PlGF) and the antiangiogenic factor sFlt-1 are balanced, maintaining normal placental angiogenesis and development [24]. In pregnancies complicated by severe PE, PlGF levels are very low, and sFlt-1 levels are significantly increased in maternal plasma [25,26]. The sFlt-1 receptor is a non-membrane-associated, and therefore free-circulating, antiangiogenic splice variant of the vascular endothelial growth factor (VEGF) receptor 1 (or Flt-1). As sFlt-1 resembles Flt-1 in its extracellular domain, it can compete with Flt-1 by binding to angiogenic factors, such as VEGF and PlGF, thereby removing them from circulation and diminishing their angiogenic effects [27]. In their innovative work, Spradley, Karumanchi et al. provided a pathophysiological pathway for placental physiology and endothelial cell/angiogenic dysfunction, which they attribute to the cause of PE. They showed in a rat model of PE that placental ischemia and hypoxia lead to sFlt-1 overexpression and elevated blood pressure, and that PlGF infusion could rescue the phenotype [25]. Collectively, this work ultimately led to several clinical trials testing whether the PlGF-to-Flt-1 ratio test or low PlGF levels can predict PE [26,28]. Reduced first-trimester PlGF and sFlt-1 were also noted in women with preexisting cHTN, another high-risk condition for developing PE. Notably, this PlGF Flt-1 ratio was more pronounced in women who developed PE w/o preexisting cHTN [24].

The Triage^®^ PlGF test [29] and the sFlt1:PlGF ratio test from Roche Diagnostics are currently in clinical use in numerous high-income countries worldwide and have been shown to have good sensitivity and predictive values for adverse pregnancy outcomes, including preeclampsia, FGR, and PTB [30]. Although these tests show great promise as a valuable tool in the second trimester for identifying the risk of developing PE within the next two weeks [31], they do not accurately predict early PE during the first trimester, when prevention is available, as sFlt-1 does not increase substantially in PE pregnancies until 18–20 wga [30].

### 5.2. Maternal Cell-Free DNA

A promising study by Adil et al. recently proposed a prediction model for PE that quantifies maternal and fetal tissue signatures using nucleosome accessibility, revealing early placental and endothelial dysfunction. They analyzed the whole genome of prenatal maternal cell-free DNA obtained as first-trimester screening for aneuploidy. They reported a validation performance of 0.85 area under the receiver operating characteristic curve (AUC, with 0.81 sensitivity at 80% specificity) for preterm PE several months before disease onset in a separate cohort of 831 consecutively collected samples. Subsequently, they confirmed this finding in an external cohort of 141 samples (AUC 0.84, 79% sensitivity). They concluded that their study paves the way for more extensive research to validate the clinical utility of this method prospectively [31]. One limitation of this study is that it does not explain the underlying causes of PE or elucidate its etiology.

### 5.3. Doppler of the Uterine Artery

Doppler US of the uterine artery provides a non-invasive method for assessing uteroplacental circulation. Several studies have demonstrated a significant decrease in spiral arterial resistance during the first trimester of pregnancy. The observation of an abnormal Doppler flow velocity waveform constitutes indirect evidence of abnormal placentation, PE, FGR [32,33], placental abruptio, and fetal demise. Indeed, the uterine artery pulsatility index (UtA-PI) is a promising screening tool for PE, particularly for early-onset PE, when combined with other biomarkers. A meta-analysis of 18 studies with 55,974 patients demonstrated that the specificity and sensitivity of abnormal UtA-PI alone for predicting early-onset PE were 47.8% (95% CI, 39–56.8%) and 92.1% (95% CI, 88.6–94.6%), respectively [34]. A major limitation of this modality stems from inter-sonographer differences despite the availability of prescriptive protocols [35,36].

#### Risk-Scoring Systems to Screen High-Risk Populations for PE

Early intervention during the first trimester in high-risk populations is critical, underscoring the need for early, accurate screening for high-risk populations. The most frequently used risk-scoring systems are those proposed by the American College of Obstetricians and Gynecologists (ACOG) and the National Institute for Health and Care Excellence (NICE). Both are based on a series of risk factors derived from maternal characteristics and medical history. However, evidence indicates that PE screening based solely on these characteristics is suboptimal. For example, when using the ACOG criteria the detection rates for early PE, preterm PE and all PE was 54.3% (95% CI, 37.0–71.6%), 65.9% (95% CI, 55.4–76.4%) and 67.1% (95% CI, 55.4–76.4%), respectively, while NICE criteria the detection rate for early PE, preterm PE and all PE was 40.2% (95% CI, 22.7–57.7%), 46.7% (95% CI, 35.3–58.0%) and 43.7% (95% CI, 37.4–50.0%), respectively [37].

The Fetal Medicine Foundation (FMF) proposed an integrated algorithm that improved prediction by combining multiple variables, including maternal characteristics, mean arterial pressure, UtA-PI Doppler, and biomarkers (PAPP-A and PlGF). According to this model, the prediction rates for very early PE (<32 wga), preterm PE, and term PE were 90%, 75%, and 41%, respectively [38].

Although the FMF prediction model shows promise, it requires the use of advanced equipment, experienced sonographers, PlGF testing, and quality control assessment, which are neither feasible nor widely available in most parts of the US or in most parts of the world, particularly in rural, low-resource settings [36], and limit its use to high-resource settings [28,39,40]. The current ACOG recommendations state that biomarkers and UA Doppler ultrasound should remain investigational [11] and emphasize that the need for simple, cost-effective early biomarkers is still at its peak.

## 6. Prevention of PE with Therapeutic Agents

Early preventive intervention may alter the course of PE and has led to a growing interest in identifying new therapeutic agents for its prevention or treatment. Indeed, there is an increase in the search for new candidate drugs that could affect the disease’s underlying pathophysiology.

Aspirin, an acetylsalicylic acid and a non-steroidal anti-inflammatory drug, is the only drug currently used in PE prophylaxis. At doses below 300 mg, it irreversibly inactivates cyclooxygenase-1, reducing prostaglandin and thromboxane production, and thereby suppressing inflammation and platelet aggregation. Emerging evidence indicates that inadequate trophoblastic invasion in PE causes an imbalance between angiogenic and antiangiogenic proteins, leading to widespread inflammation, endothelial damage, heightened platelet aggregation, and thrombotic events such as placental infarcts, which are characteristic of PE [41]. This has led to the hypothesis that Aspirin may help prevent PE. Randomized trials testing this hypothesis yielded contradictory findings, primarily due to significant heterogeneity in PE definitions, participant selection, baseline risks among women, aspirin dosage, and gestational age at the initiation of prophylaxis. A 2007 meta-analysis by Askie et al. reported a modest 10% reduction in PE with aspirin treatment (RR 0.9; 95% CI 0.84–0.97) and noted significant heterogeneity among the included studies [40]. A series of subsequent meta-analyses have revealed that Aspirin is highly effective if initiated before 16 wga (RR, 0.47; CI, 0.34–0.65) [42] but confers no benefits when started after 16 wga (RR, 0.11; CI, 0.04–0.33), no significant beneficial effect on term PE (RR, 0.98%; CI, 0.42–2.33) [42,43], and there is a dose–response effect when Aspirin is initiated before 16 wga [43,44].

The Aspirin for Evidence-Based Preeclampsia Prevention (ASPRE), a multicenter, double-blind, placebo-controlled trial, evaluated Aspirin at a dose of 150 mg, initiated at 11–14 wga and continued until 36 wga, in high-risk populations, and its effect on preterm PE (<37 wga). High-risk women were selected using the FMF-proposed integrated algorithm, as described above. Eventually, 1776 high-risk women were recruited, and treatment with Aspirin was found to reduce the rate of preterm PE by 62% (1.6% vs. 4.3%; OR, 0.38%; 95% CI, 0.2–0.74; *p* = 0.04). At 90% compliance, the effect size of Aspirin was even larger at 76% and could reach 90% when no prior cHTN was present [22]. There was no significant decrease in the rate of term PE. Despite this, the authors reported no significant differences in the incidence of neonatal adverse outcomes between groups. The conclusion of the ASPRE study is to start high-dose Aspirin between 11–14 wga for optimal PE prevention. A study limitation originates from the prevalence of preterm PE in the placebo group, which was one-half of that expected for the high-risk population based on first-trimester parameters [22].

The ACOG guidelines, on the other hand, recommend low-dose Aspirin (LDA) prophylaxis at 81 mg/day in women at high risk of PE, to be initiated between 12 and 28 wga (optimally before 16 weeks) and continued daily until delivery [11].

The benefit of early initiation of Aspirin during pregnancy to prevent PE supports the hypothesis that excessive, ongoing early second-trimester inflammation at the maternal–fetal interface contributes to the development of PE. While the first trimester of implantation and the early second-trimester placental development constitute an autoimmune anti-inflammatory phase, by 16 weeks of gestation in the mid-second-trimester, maternal immune cells in the decidua should have adapted to a more tolerant Th2 phenotype [45]. Interestingly, a recent study showed that LDA administered from early pregnancy through the third trimester to women at high risk of PE increased CD4+ and CD8+ cells, naïve Treg cells, and shifted NK cells from cytotoxic to tolerogenic in the peripheral blood. This was accompanied by a decrease in activated inflammatory Th17 cells, indicating enhanced immune tolerance and reduced inflammation, supporting Aspirin’s role in modulating immune responses to promote normal pregnancy outcomes [46]. Still, the specific effect of aspirin on decidual immunology is currently unknown.

Other new therapeutic agents, such as Statins, Metformin, Proton pump inhibitors, and Sulfasalazine, have been widely investigated. Still, current evidence is insufficient to establish a definitive protective role in preventing preeclampsia during pregnancy.

In summary, although there has been an impressive increase in clinical trials of new drug candidates for the treatment or prevention of disease, there remains an imperative need for large, global, randomized studies to advance the field.

## 7. The Physiology of the Maternal–Fetal Interface in Healthy Pregnancy and in PE

**Figure 1:**
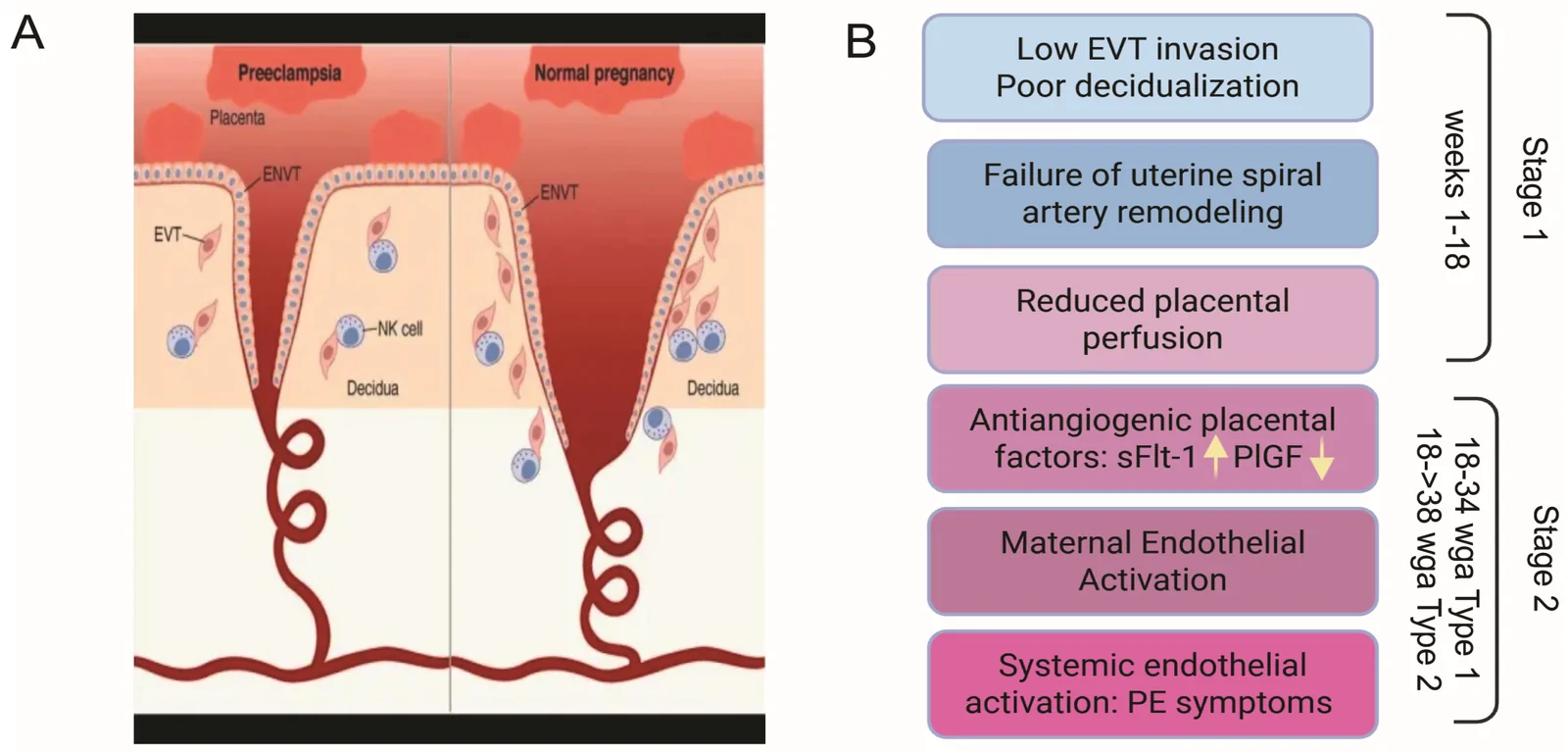
Failure of vascular remodeling in PE and the 2-stage theory of PE onset. (**A**) In the preeclamptic placental bed (Left), the transformation of the uterine spiral arteries is limited to the decidua, and the number of EVT (pink) cells is lower. This results in pulsatile high-pressure blood flow and damage to the placenta. In a healthy pregnancy (Right), the ENVT that lines the vessel invades as far as the first third of the myometrium (white), leading to a large increase in low-pressure blood flow to the placenta. EVT directly interacts with the decidual NK (blue) cells in this process. (**B**) The 2-stage theory of sequential steps leading to the onset of PE symptoms in the mother.

Establishment of the maternal–fetal interface begins early in pregnancy between days 5 and 6 following fertilization, as soon as the blastocyst, consisting of an inner cell mass (which will become the embryo) and an outer layer of cells (which will develop into the placenta), attaches and implants into the uterine lining, also known as the decidua. This outer cell layer, termed the trophectoderm, is the precursor of all trophoblast populations of the placenta [47]. These cells proliferate rapidly to form a multilayered trophoblast shell. Within this shell, the early differentiation of the villous placenta begins, while the outer layers generate the invasive extravillous trophoblast (EVT) that migrates into the decidua [48]. EVTs are found in the interstitial areas of the decidua and are associated with veins and glands, potentially playing a role in the embryo’s early histotrophic nutrition before blood flow begins [48]. By 7 to 8 wga, many of the major organ systems in the fetus have begun developing, and the placenta shifts from a circle of villi surrounding the fetus into a flat disc attached to the decidua by finger-like villous projections. Within the placenta, a richly branched fetoplacental vascular network begins to form within the placental villi, which fuses with the fetal allantois, which will become the umbilical cord. At the tips of these villi, anchoring cell columns form, serving as the origin of invasive EVT that intermingles and penetrates the inner maternal uterine layer, the decidua. The EVTs also penetrate the uterine spiral arteries and transform them by removing the arterial smooth muscle wall and relining the vessel with endovascular EVT (ENVT), as shown in Figure 1.

These changes significantly increase the maternal blood volume in the intervillous space and at the placental surface [49,50]. Blood flow begins between 10–12 wga in both the fetal-placental and the utero-placental circulations, which carry maternal blood to the intervillous space and supply the placental surface. Maternal and fetal blood are separated by the specialized, fused outer syncytiotrophoblast layer of the placental villi.

The transformation of the deeper myometrial portions of the decidual spiral artery is critical for establishing the high-flow, low-pressure circulation of the intervillous space, which facilitates the exchange of nutrients, waste products, and gases between the mother and the fetus [49,51]. This process of utero-vascular remodeling continues into the late second trimester as the EVT invades as far as the first third of the myometrium. In a healthy pregnancy, approximately 100–150 uterine spiral arteries supply the intervillous space, and by term, blood flow to the intervillous space is estimated at 400–700 mL per minute [52]. The maternal heart and cardiovascular system also undergo extensive remodeling, and the left ventricle and the aorta enlarge to increase cardiac output and adapt to the increased maternal blood flow to the placenta and fetus [53].

The failure of transformation of the uterine spiral arteries that supply the intervillous space and surface of the placenta with maternal blood is thought to contribute to several disorders of pregnancy, including PE [54,55,56]. These cases exhibit decreased or pulsatile utero-placental blood flow, indicated by persistent high-resistance waveforms on color and pulsed Doppler ultrasound of the proximal uterine spiral arteries [57,58]. Placental bed biopsies from these subjects at delivery show failure of ENVT invasion into the myometrial portions of spiral arteries despite adequate decidual interstitial EVT invasion [59,60]. It is thought that reduced, pulsatile blood flow to the placenta results in placental damage and the second stage of PE, and the release of vasoactive factors that lead to the activation of the maternal systemic endothelium and the onset of PE symptoms and signs in the mother (Figure 1) [61].

## 8. The Immunology of the Maternal–Fetal Interface

The immune-maternal adaptation to paternal foreign-derived antigens, whereby they are perceived as “self,” is a unique immunological phenomenon that occurs during pregnancy. Immunological adaptations are required at both the maternal/decidual and fetal/placental interfaces to enable this process and are described below.

### 8.1. The Immunology of the Maternal Decidual Interface

**Figure 2:**
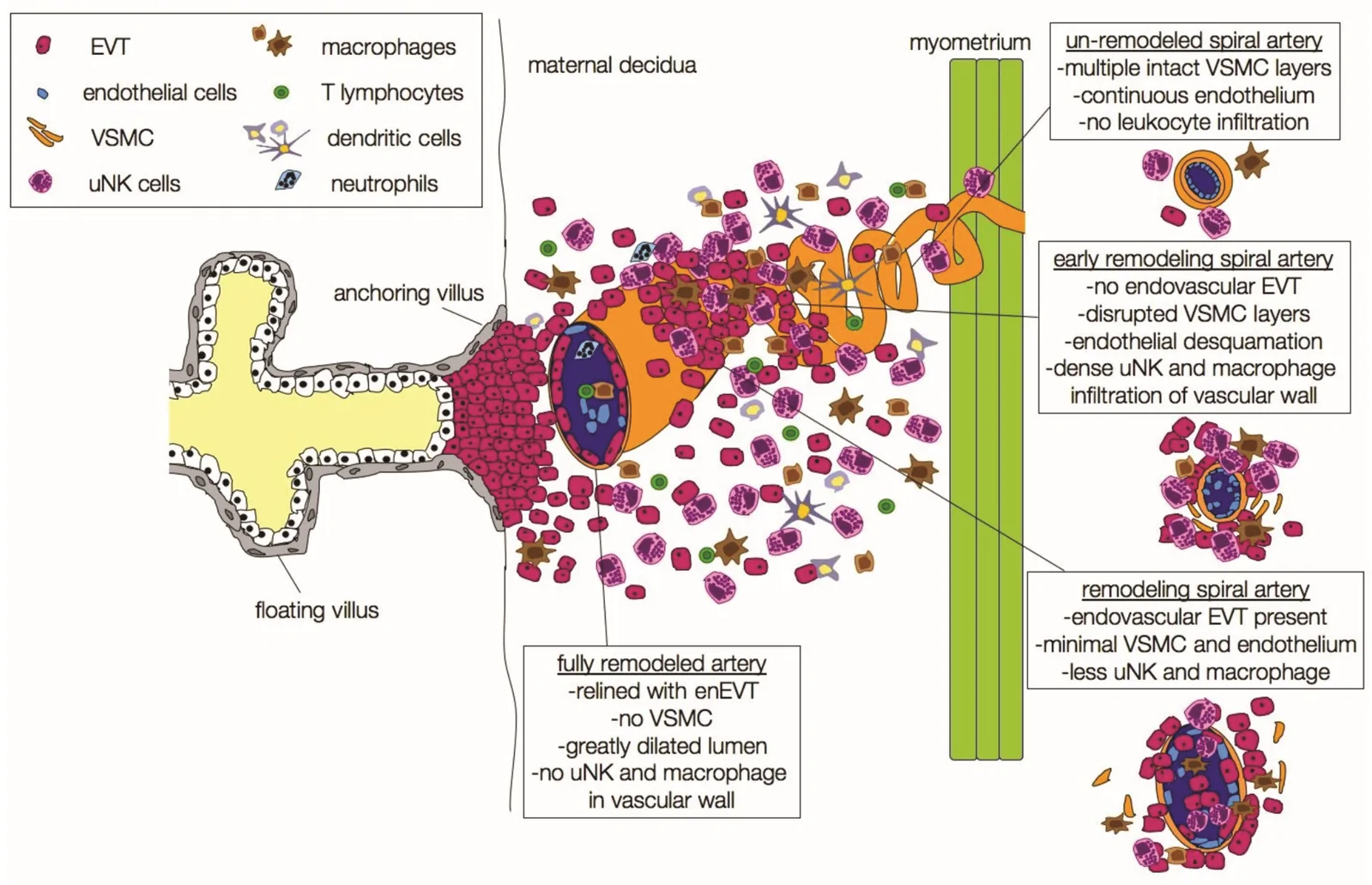
Sequential stages of vascular transformation mediated by EVT, dNK, and macrophage cells. EVT-secreted chemokines activate the vascular endothelium, which then recruits dNK and macrophages into the vascular wall, where they secrete matrix metalloproteinases to disrupt the vascular smooth muscle, leading to vessel widening and relining by ENVT. Decidual T cells, dendritic cells, and neutrophils are regulatory cells and are proangiogenic and tissue remodeling in phenotype. Created in BioRender. Wright, T. (2026) https://BioRender.com/re8foxv (accessed on 7 April 2026).

Immune cells comprise approximately 40% of cells in the decidua, and their number is highest during the first trimester following conception [62]. The specialized tissue-resident maternal decidual cells comprise white blood cells, macrophages, lymphocytes (including T cells), myeloid-derived suppressor cells (MDSCs), and decidual natural killer (dNK) cells, which are known for their unique properties (Figure 2).

#### 8.1.1. dNK

The dNK cells comprise 50–70% of decidual lymphocytes [63] and are in direct contact with extravillous trophoblast cells and spiral arteries [64]. In humans, the number of dNK cells decreases steadily from the early second trimester, suggesting their central role in placentation and spiral artery remodeling during the early first trimester. Indeed, a seminal experiment found that pregnancies in mice lacking dNK cells resulted in defective remodeling of the central artery and growth-restricted pups [65].

Although peripheral blood NK (pbNK) cells in maternal circulation are known for their potent cytotoxic effects against foreign cells and pathogens, dNK cells in the decidua produce chemokines, growth factors, cytokines, and angiogenic factors essential for proper placental bed development during implantation [63]. It is well established that dNK cells secrete interleukin-8 and interferon gamma-induced protein 10 to stimulate EVT invasion. Reciprocally, the EVT expresses the chemokine (CXCL12), activating its receptor CXCR4 on dNK cells. This bidirectional chemokine signaling facilitates the colocalization of EVT and dNK within the decidua [66].

Phenotypically, dNK cells are similar to a minority population of pbNK cells, characterized as CD56brightCD16neg, suggesting that dNK cells likely develop from a subset of pbNK cells that migrate from the blood to the decidua [63]. Several recent studies have shown considerable heterogeneity in dNK populations: single-cell sequencing and mass cytometry experiments have identified 3 distinct NK populations, each expressing distinct cell surface markers, transcription factors, and receptors, as well as a 4th proliferating phenotype. The dNK2 and dNK3 subsets produce more inflammatory chemokines than dNK1 when stimulated and can act on both maternal dendritic cells and fetal EVT [67,68,69]. A recent single-cell sequencing study using human EVT organoids cocultured with freshly isolated decidual NKs further suggested that each dNK population—1/,2/, or 3—may interact with or stimulate the differentiation of EVT into three distinct classes each, separated by distinct gene expression profiles associated with epithelial-to-mesenchymal transition and EVT differentiation and invasion [70]. More recently, Li and colleagues used their single-cell sequencing data, which originally identified the 3 NK subtypes, to identify a restricted set of cytokines—CSF1, CSF2, XCL1, and CCL5—that are expressed exclusively by dNK cells and whose receptors are expressed by EVTs. Using the trophoblast organoid model, they show that a cocktail of these 4 cytokines recapitulates the EVT differentiation pathway observed in vivo, resulting in an increased proportion of late EVTs, resembling the interstitial EVT found deep in the decidual stroma [71].

The dNK cells also play a crucial role in the remodeling of uterine spiral arteries, as discussed above. Along with macrophages, dNK cells accumulate around decidual arteries and secrete matrix metalloproteinases that degrade the extracellular matrix of vascular smooth muscle cells, leading to their dedifferentiation and vessel dilation [50,72,73]. This immune-mediated vascular transformation process is contingent on the presence of the EVT, which initially activates the vessels by secreting interleukin (IL)-6 and CXCL8 and induces endothelial cell secretion of chemokines CCL14 and CXCL16, leading to further recruitment of dNK and macrophages into the vessel wall [74]. Subsequently, the endovascular EVT invasion of the vessel lumen is then stimulated by dNK and macrophages in the vessel wall, and it is thought to progress upward along the vessel into the myometrial junction (Figure 2).

#### 8.1.2. The dNK Receptors

The education of NK cells and their response to infection, cancer, and allogeneic tissue are guided by interactions between NK cell receptors and MHC class I ligands. NK cells recognize their targets through a variety of inhibitory and activating receptors [75], and most NK inhibitory receptors bind MHC class I [76]. These engagements enable NK cells to distinguish diseased cells, whose MHC class I expression is perturbed, from normal healthy cells [77].

Leukocyte immunoglobulin-like receptor 1 (LILRB1), a member of the leukocyte immunoglobulin-like receptor (LIR) family, is one of the NK inhibitory receptor family [78] that recognizes a broad spectrum of major histocompatibility complex (MHC) class I molecules, with a preference for HLA-G binding. LILRB1 and LILRB2 receptors are also expressed on monocytes and dendritic cells. Their binding to the α3 domain of HLA-G transduces inhibitory signals via immune tyrosine-based inhibition (ITIM) motifs, dampening immune responses and promoting tolerance. LILRB1 engagement reduces cytotoxicity in decidual NK and T cells, while LILRB2 induces a regulatory macrophage phenotype [79].

dNK cells express most of the NKG2 activating and inhibitory receptors expressed by pbNK cells [80], including NKG2C and NKG2A receptors, which recognize the placental non-classical MHC class I protein. NKG2A contains two ITIMs in its cytoplasmic tail that send inhibitory signals to cytolytic NK cells. Unlike its inhibitory counterpart, NKG2A, NKG2C lacks an intrinsic signaling domain. Instead, it is expressed with DAP12, which carries the immune receptor Tyrosine-based activation Motif (ITAM) that initiates activation signals. Cell surface NKG2A and NKG2C are dependent on a covalent disulfide bond with CD94, a protein expressed on NK cells and a subset of T cells, which is essential for transporting them to the cell surface [81]. Both CD94/NKG2A and CD94/NKG2C bind HLA-E, which is expressed on EVTs. Notably, some studies indicate that CD94/NKG2A preferentially binds HLA-E with approximately sixfold higher affinity than CD94/NKG2C [82,83,84].

Killer cell immunoglobulin-like receptors (KIRs) are also important receptors on pbNK and T cells for self-tolerance, mediating cytotoxic activity against cancer cells and virus-infected cells. The KIRs are transmembrane glycoproteins that are highly polymorphic and exist in numerous short and long forms; they bind to classical MHC class I antigens and can have either inhibitory or excitatory capabilities. These receptors are also expressed on dNK and interact with fetal HLA-C and HLA-F [85].

An additional class of receptors expressed by dNK cells includes Natural Cytotoxicity, consisting of the NKp (NK protein) receptors, which are major activating receptors. Naive dNK cells express the NKp receptor NKp44, which is found only in pbNK cells after activation [64,86]. In a study comparing first- and second-trimester dNK, Zhang and colleagues showed that while the number of dNK cells expressing Nkp44 was low, most dNK cells expressed both Nkp46 and Nkp30 [87].

The dNK also differs from pbNK by expressing receptors absent in pbNK cells, and the functions of some receptors in dNK cells differ from those in pbNK cells. For example, LILRB1 is an inhibitory receptor on pbNK cells, whereas in the decidua, it functions as an activating receptor [88,89]. Furthermore, 2B4, which serves as a co-activating receptor in human pbNK cells, functions as an inhibitory receptor in the decidua [10,90]. Finally, some inhibitory receptors have activating counterparts. As discussed above, dNK cells express a heterodimer receptor composed of CD94/NKG2A that recognizes the non-classical placental MHC class I protein HLA-E and acts as a key inhibitory receptor [91]. In contrast, when the CD94 heterodimerizes with NKG2C, it activates NK cells upon interaction with HLA-E, although the interaction is low-affinity [91]. For a comprehensive review of receptors expressed by dNK, please see the references [87,92].

#### 8.1.3. Memory dNK

**Figure 3:**
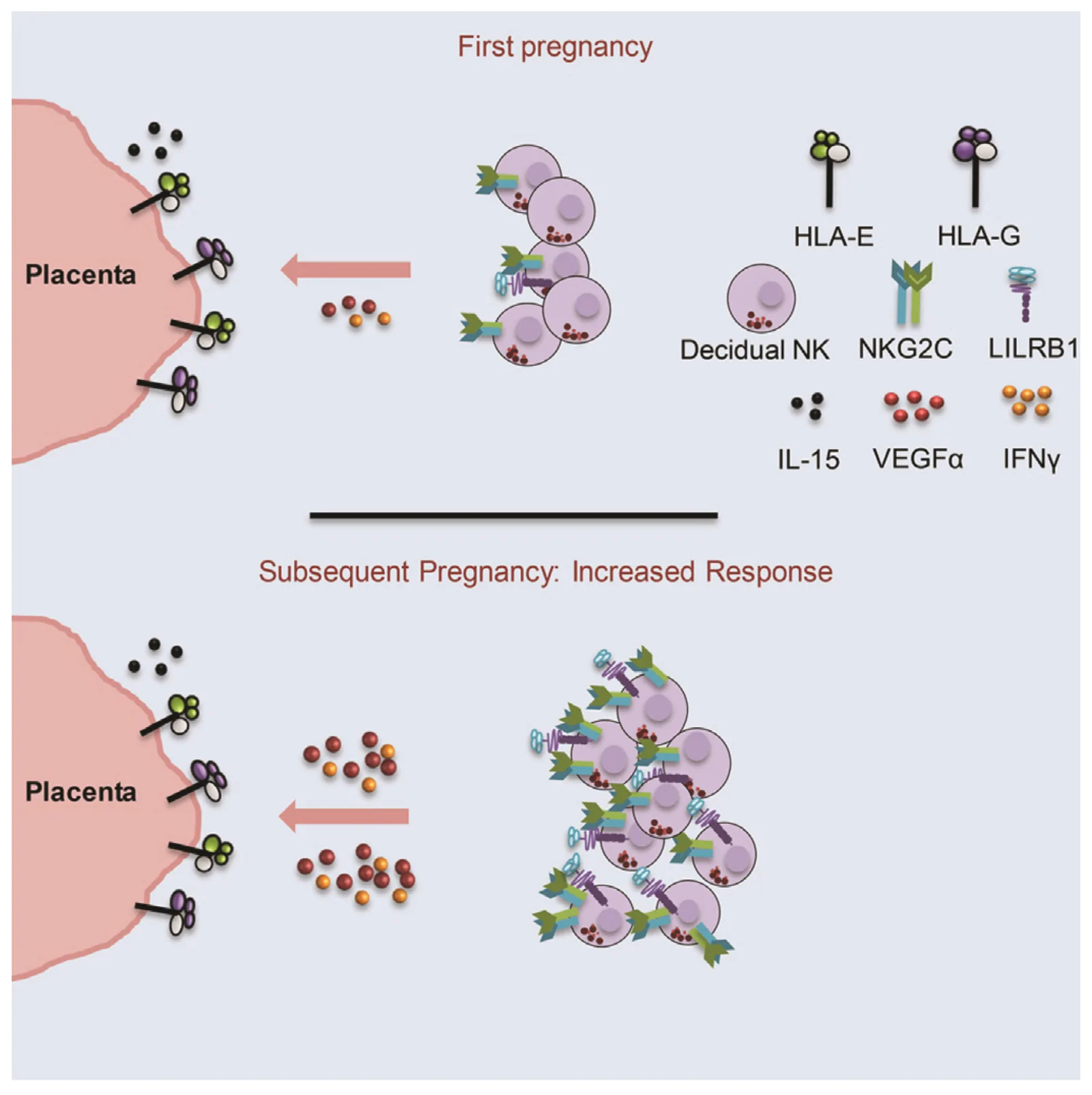
Memory NK cells. In multigravida women, the dNK cells express high levels of the NKG2C and LILRB1 receptors that bind EVT-expressed HLA-G and HLA-E. They are termed pregnancy-trained dNK and have a unique transcriptome and secrete higher levels of the angiogenic and inflammatory proteins VEGF-A, IFN-γ, and IL-15 that act on the placenta via their receptors (arrows). Reproduced with permission from Immunity Journal [10].

In their recent groundbreaking work, Gamliel et al. demonstrated that human and mouse NK cells possess memory-like properties. They characterized dNK in both first and repeat pregnancies. They identified a unique transcriptomic and epigenomic signature in parous women. They named these cells pregnancy-trained dNK (PTDdNK), which are primed to act with greater expression of the receptors NKG2C and LLRB1, which bind to placental HLA-G and HLA-E. These features are stored as epigenetic marks in NK cells residing in the endometrium between pregnancies, where they remain “on call” to respond more robustly in subsequent pregnancies. Indeed, PTdNK showed increased secretion of IFN-γ and VEGF-A upon NKG2C interaction with HLA-E and LILRB1 interaction with HLA-G. The VEGF-A obtained from multigravida (but not primigravida) was functional, as demonstrated by ex vivo aortic sprouting and in vivo tumor growth models. This suggests that PTdNKs can better support vascularization, a critical component of placental bed development. They also confirmed that LILRB1 functions as an activating receptor in the decidua [10]. Activation of this receptor on PTdNKs by HLA-G (uniquely expressed on EVT cells) in the presence of IL-15 led to increased secretion of VEGF-A and IFN-γ, as shown in previous research (Figure 3) [89]. This enhanced activation of interleukins, hormonal regulation, and chemokine ligands and receptors may remodel the placental bed more efficiently, resulting in improved placentation and a lower risk of developing PE in multigravida, as shown in demographic studies [10,93,94]. A major limitation of this study is that it was performed in mice and ex vivo, and not in human pregnancy.

This research provides a biological explanation of the higher rate of PE in primigravidae, attributed to suboptimal implantation and uterine adaptation, secondary to a new immunological exposure as opposed to multigravidae [10]. These findings further support the critical immunological role in ensuring healthy pregnancies [95].

#### 8.1.4. Myeloid-Derived Suppressor Cells (MDSCs)

MDSCs are a heterogeneous cell population consisting of immature granulocytes, macrophages, and dendritic cells that have recently been associated with successful implantation and pregnancy outcome. MDSCs tend to increase during pregnancy in the peripheral blood, bone marrow, and uterus. Decreased levels are associated with miscarriages and PE. The immunosuppressive function of MDSCs remains investigational, and they exert their inhibitory effects through multiple mechanisms. MDSCs modulate dNK and Treg by increasing their suppressive cytokine activity via IL-10 production. MDSCs also efficiently suppress T cell proliferation by expressing ARG-1 and iNOS and producing high levels of ROS. In bone marrow, cytokines such as GM-CSF, M-CSF, IL-1, IL-6, IL-10, IL-12, and IL-13 are involved in their maturation. In the maternal periphery, factors such as CXCL5, CXCL12, and S100A8 and S100A9 may help recruit MDSCs to the maternal–fetal interface. Importantly, MDSCs also have non-immunologic functions such as angiogenesis [95,96].

### 8.2. The Immunology of the Fetal Placental Interface

A significant genetic determinant of both the general and specific immune systems during pregnancy is contained within the major histocompatibility Complex (MHC) [97]. Expression of MHC genes determines self- and non-self-recognition in the human immune system. In humans, this region is located on the short arm of chromosome 6 and includes the classical class I molecules, which are expressed by all nucleated cells. The role of these molecules in immunological recognition is now well understood [98]. The MHC also contains three highly homologous, non-classical class I genes, HLA-E, F, and G, which are expressed by placental cells, including EVT. The non-classical HLA gene locus was initially identified in 1988 by Koller, Geraghty et al. [99]. Unlike the classical HLA, the non-classical HLA antigens (NCHLA) are low polymorphic and, as such, are thought to provoke less immunological reaction.

The human placenta is the only organ that expresses all three non-classical HLAs and the polymorphic classical HLA-C, supporting the concept of the placenta as an immune-privileged organ [100]. Indeed, since the discovery of unique placental HLA expression [99,100,101], numerous studies have explored its role in pregnancy, including in vitro fertilization (IVF) pregnancy, and in the very early stages of pregnancy.

In vitro studies have shown interactions between NCHLA and dNKG2C, NKG2A, LILRB1/2, and KIRs at the decidual-placental interface [102,103,104]. Interestingly, NCHLA can also bind to each other, forming combined peptides and tetrameric HLA complexes that interact specifically with maternal decidual NK, KIR, and LILRB1/2 receptors [8,10,102].

In this section, we will review relevant information on non-classical placental HLA and placental HLA-C and further discuss their interrelation with the decidual interface.

#### 8.2.1. HLA-G

**Figure 4:**
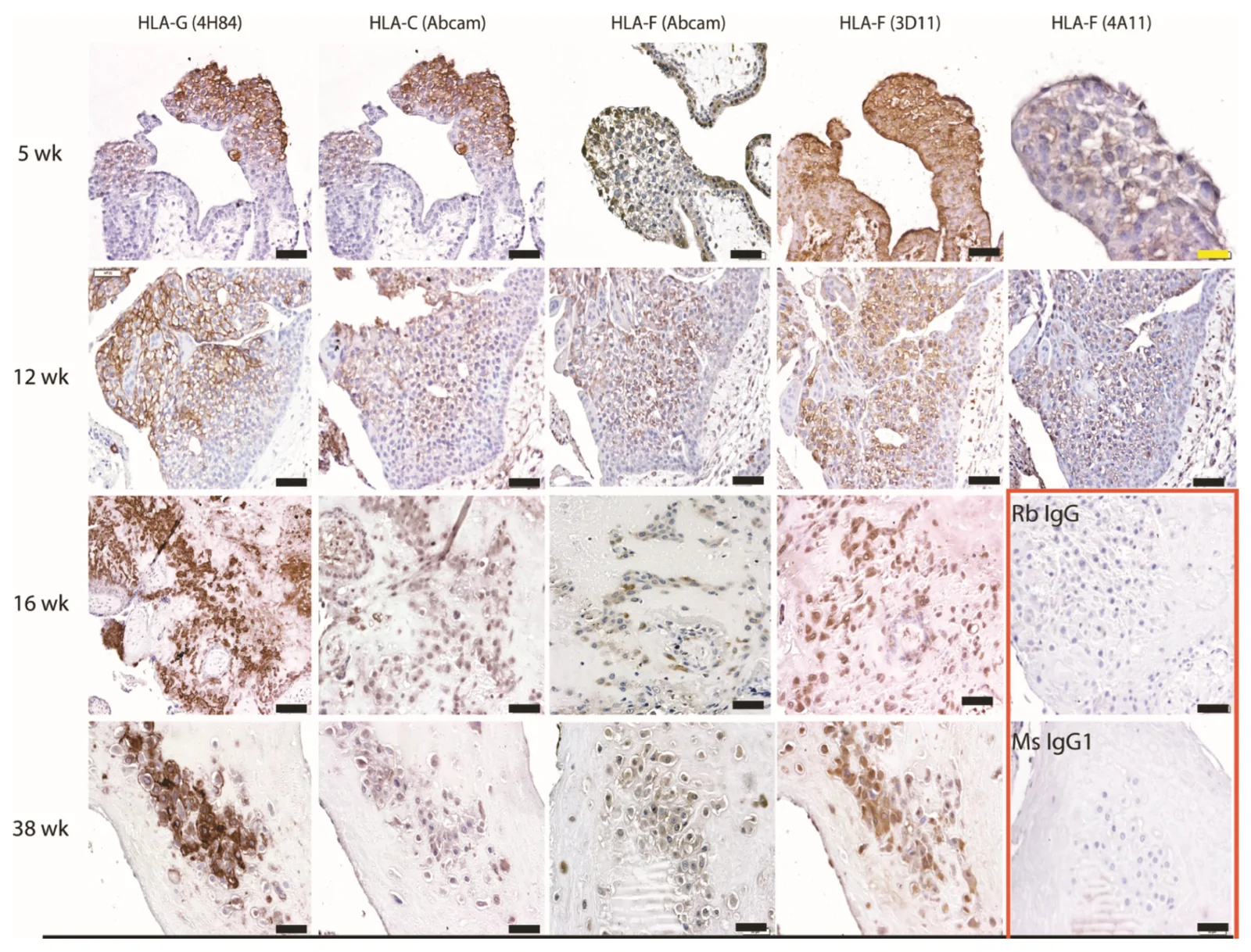
Immunolocalization and expression profile of HLA-F and HLA-C in EVT across gestation. Representative photographs of EVT in serial sections of anchoring columns in 5-wk and 12-wk placenta and interstitial EVT within 16-wk and 38-wk decidua. The EVT populations are marked by brown staining for HLA-G (4H84) in the first column. HLA-C polyclonal antibody staining is shown in the 2nd column; HLA-C is expressed in the membranes of early EVT and is cytoplasmic at later gestational ages. The HLA-F (rabbit monoclonal Abcam, 3rd column) and 4A11 (mouse monoclonal, 5th column) show strong expression in the membranes of 1st-trimester EVT. Cytoplasmic staining of EVT for HLA-F (3D11 mouse monoclonal, 4th column) was seen at all gestational ages. Negative controls were performed using non-immune mouse IgG1 (MsIgG1 at 1 μg/mL) or rabbit IgG (RbIgG at 4.5 μg/mL). n = 5 at each gestational time point; yellow bars = 20 μM, black bars = 50 μM. Modified and reproduced with permission from AJRI [100].

The HLA-G gene and its antigen are among the most thoroughly researched non-classical HLAs. It consists of eight exons and seven introns; exons 2–4 encode the extracellular domains involved in antigen presentation and receptor binding, while exon 8 encodes the 3′ untranslated region (3′UTR), which is vital for controlling mRNA stability and translation. HLA-G generally forms a non-covalent complex with β2-microglobulin, which is crucial for its stability and function. HLA-G is primarily expressed in immune-privileged sites such as the placenta, as well as in specific tissues including the testis, prostate, cornea, thymus epithelial cells, retinal pigment epithelial cells, decidual stromal cells, and pancreas [105,106,107,108,109,110,111]. In the placenta, HLA-G expression is restricted to the more distal, fully differentiated EVT of the anchoring cell columns and the invasive EVT found in the decidua basalis. Interestingly, HLA-G is most strongly expressed on the cell surface in early first-trimester EVT and gradually transitions to lower expression levels, becoming mostly cytoplasmic by the second trimester, when the EVT are within the placental bed (Figure 4) [100].

Notably, HLA-G can enhance the surface expression of other non-classical HLA molecules, such as HLA-E, in target cells [112], which, through the CD94/NKG2A and CD94/NKG2C receptors, can also exert inhibitory and excitatory effects on NK cells, respectively [113].

HLA-G uniquely possesses several splice variants, including four membrane-bound proteins (HLA-G1 to HLA-G4) and three soluble proteins (HLA-G5 to HLA-G7), all of which are expressed by the EVT [114]. Soluble forms of HLA-G are secreted into extracellular fluids, including follicular fluid, embryonal culture media, maternal serum, and amniotic fluid [115]. Soluble HLA-G can bind to and be internalized by LILRB1 and LILRB2 and may contribute to maternal immune memory in subsequent pregnancies. The soluble isoforms play critical roles in embryo-maternal communication [116], and their levels are associated with increased implantation and pregnancy success in IVF. In a pioneering study by Fuzzi et al. detectable sHLA-G in supernatants from cultured embryos was associated with a higher likelihood of achieving pregnancy after IVF/ICSI [117]. A subsequent study reproduced this result [118]. These studies have shown that, early in the blastocyst stage, non-classical HLA forms are already expressed and associated with fertilization and implantation rates. These findings were supported by another study, which also indicated that embryos producing sHLA-G in the IVF culture supernatant are more likely to develop into blastocysts [118]. Furthermore, sHLA-G has been shown to be present in the male reproductive system, primarily in the epididymis, testes, and seminal plasma, suggesting a role in male fertility prior to pregnancy as well [109,111].

#### 8.2.2. HLA-E

Unlike the restricted HLA-G cell expression, the HLA-E antigen is ubiquitously expressed on all nucleated cells [119]. Structurally, the HLA-E antigen forms a heterodimer composed of a membrane-bound α heavy chain (~45 kDa) and a β2-microglobulin light chain. Its gene consists of seven exons: exons 1–4 encode the extracellular domains (α1, α2, α3), exon 5 the transmembrane region, and exons six and 7 the cytoplasmic tail. Despite this genomic structure, exons 7 and 8 are typically excluded from the mature mRNA transcript, resulting in a shortened cytoplasmic tail [120].

HLA-E is distinct in that it requires peptides derived from the leading sequence of other HLA-A, HLA-B, and HLA-C, as well as HLA-G, for appropriate folding and transport to the cell surface via transporter-associated antigen presentation (TAP) [84,113]. These leader peptides also stabilize the HLA-E antigen at the cell surface and facilitate its interaction with T cells. Due to its restricted peptide repertoire and conserved anchor residues, HLA-E’s function is more immunoregulatory than antigen-presenting [121].

**Figure 5:**
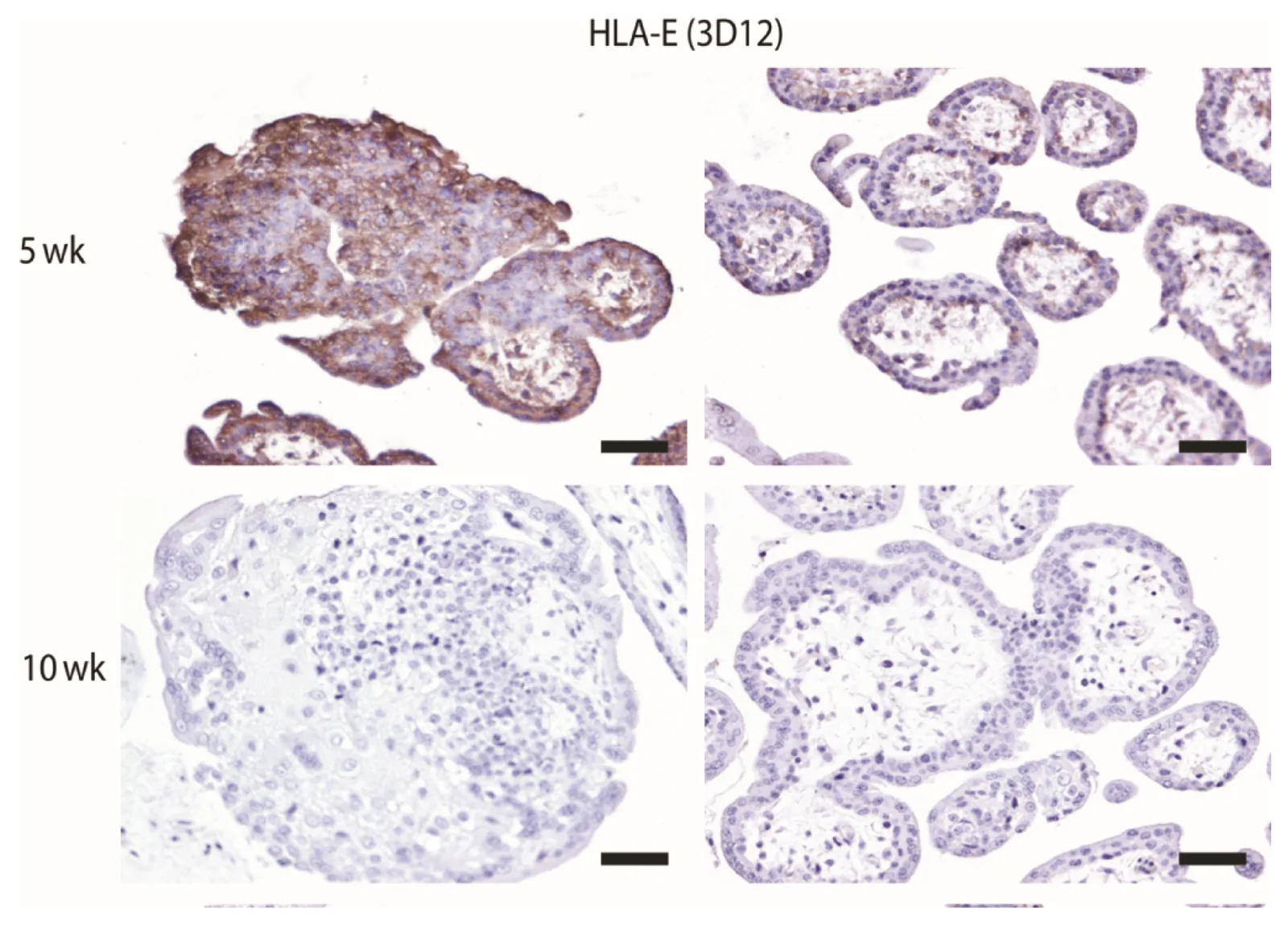
HLA-E expression is restricted to the early 1st-trimester placenta. Representative photographs of placental anchoring villi with EVT (1st column) and placental villi (2nd column) from 5 and 10 weeks. HLA-E is expressed in EVT at 5 weeks’ gestation and also in the trophoblast bilayer (brown staining). HLA-E is not detected from 10 weeks onwards. n = 5. Black bars = 50 μM. Modified and reproduced with permission from AJRI [100].

In the placenta, where no HLA-A or HLA-B are expressed, HLA-E preferentially presents leader peptides of HLA-G [122]. The peptide VMAPRTLFL, originating from HLA-G, forms a complex with HLA-E and promotes the enrichment of adaptive NK cells that show low FcεRγ levels, higher CD25 expression, enhanced proliferation, and increased antibody-dependent cellular cytotoxicity and IFN-γ responses compared to other HLA-E peptide complexes. These adaptive NK cells can precisely distinguish peptides bound to HLA-E and recognize alterations in the HLA-E ligandome via an activating receptor that can influence heterologous effector mechanisms and proliferation in adaptive NK cells [123]. It can also present noncanonical peptides to CD8+ T cells, contributing to antiviral and alloimmune surveillance [124,125]. Notably, Hackmon et al. were the first to show that HLA-E antigen expression is restricted to very early EVT in the anchoring column and is undetectable from 7 weeks onwards (Figure 5) [100]. The finding of this unique HLA-E expression in the early first trimester, at the peak of the process of immunological acceptance, led us to hypothesize that HLA-E plays an essential role in early normal placentation and pregnancy.

Indeed, IVF studies have demonstrated that dysregulated HLA-E expression may disrupt the local endometrial immune milieu, impair receptivity, and increase the risk of recurrent implantation failure (RIF). Amjadi et al. observed that endometrial samples from women with RIF show reduced HLA-E expression along with elevated levels of pro-inflammatory cytokines, e.g., IL-6 and interferon-γ (IFN-γ). This elevation of inflammatory cytokines shifts the immune environment toward a Th1-dominant state, which may prevent successful embryo implantation by limiting immune tolerance and promoting cytotoxic responses [126]. Genetic studies have further examined the association between HLA-E allelic variants and fertility. The two main coding alleles, HLA-E*01:01 and HLA-E*01:03, differ in cell surface expression and peptide-binding affinity. Notably, HLA-E*01:03 is associated with greater surface stability and stronger NK receptor binding, promoting more robust immunoregulatory effects. HLA-E*01:01, by contrast, shows reduced surface expression, which may compromise tolerance mechanisms [127]. Other studies have suggested HLA-E allelic variants—HLA-E*01:03 homozygosity in male infertility and ethnicity [127,128]. Although current evidence remains mixed and largely observational, HLA-E represents a promising target for immunogenetic screening of IVF patients, for embryo selection based on HLA-E expression in preimplantation embryos, and for personalized immunotherapy strategies in RIF and reproductive medicine [116].

HLA-E also exists as a soluble form detectable in plasma and is an emerging area of investigation as a biomarker and a marker of disease activity across various diseases. To date, much of what is known about the function and expression of sHLA-E derives from research in the cancer field, including leukemia (especially myeloid leukemia) [129], gastric cancer [130], and oral squamous cell carcinoma [131]. In infectious diseases, plasma sHLA-E (and sHLA-G) levels from COVID-19 patients have been proposed as identifiers of critical cases [132] and are associated with chronic hepatitis B (CHB) and advanced fibrosis stages. In fact, sHLA-E is such a powerful predictor of CHB progression that further research into its use as a clinical biomarker of CHB is warranted [133]. Furthermore, sHLA-E has been detected in the plasma of patients with vasculitis and suggested as a biomarker for Takayasu disease [134]. Interestingly, in vitro studies of endometrial cells showed that TNF-α, IFN-γ, and interleukin-6 upregulated the cell-surface expression of HLA-E and induced the release of sHLA-E [135]. Whether levels of sHLA-E are detectable in maternal plasma, or indeed altered in pathological pregnancies, remains unknown; still, the above studies indicate a high likelihood of detection in maternal plasma.

In summary, HLA-E’s ability to modulate NK and T cell responses at the maternal–fetal interface suggests its influence across multiple stages of reproduction, from fertilization to implantation and placental development. Its strong interaction with NKG2A and NKG2C, in particular, may be essential for fine-tuning dNK cell activity and preventing rejection of the placental-derived EVT.

#### 8.2.3. HLA-F

HLA-F antigen is expressed in various immune cells, including B cells, T cells, peripheral blood lymphocytes, and NK cells, and is inducible in activated immune environments. Its expression is predominantly intracellular under resting conditions but appears on the cell surface upon cell activation, suggesting immune state-dependent regulation [136]. Structurally similar to classical HLA class I molecules, HLA-F comprises a ~40–41 kDa α heavy chain paired with β2-microglobulin [116]. Like HLA-E, HLA-F undergoes alternative splicing that often excludes exon 7, resulting in a truncated cytoplasmic tail. This structural variation may affect intracellular trafficking and receptor signaling [137,138]. One of the unique immunological features of HLA-F is its ability to exist in an open conformer state lacking a bound peptide, which enables it to interact with various immune receptors independently of antigen presentation.

The functions of HLA-F are less well studied and understood. HLA-F is expressed on EVTs and in the thymus, as well as on endothelial cells in the tonsils, spleen, and endometrium [139]. Fertility genetic studies have identified functionally significant SNPs for HLA-F and TAP expression in the mid-secretory endometrium that are associated with shorter time-to-pregnancy [140]. One of these SNPs is located within a progesterone response element and creates a GATA2-binding site that, upon engagement, loops to HLA-F and influences expression to a degree that is dependent on the genotype [141,142].

Studies of HLA-F expression patterns in pregnancy have yielded conflicting results. One study has shown that HLA-F is expressed on the surface of EVT cells embedded in the decidua [142] as open conformers, devoid of β2m and peptide [143]. Other reports using different cell lines and primary B cells have shown that HLA-F is exclusively expressed in the cytoplasm and is associated with TAP on the cell surface [144,145]. Another study found that HLA-F is also expressed in the cytoplasm of EVT during the first trimester of pregnancy, and at the cell surface in the second trimester. Hackmon et al. found that HLA-F expression changed from the cytoplasm to the cell surface as EVT differentiated from an early proliferative to an invasive phenotype in placental explants. They validated this finding using the first-trimester EVT cell line Swan 71 and showed that the same increase in cell-surface HLA-F occurred in invasive cells migrating towards a chemoattractant. They also found cytoplasmic HLA-F in the stationary EVT of the term placental bed (Figure 4). Furthermore, quantitative studies showed that HLA-F was highly expressed and detected as a high-molecular-weight dimer or complex in first-trimester placenta and decreased in third-trimester tissues, supporting this group’s findings [100].

In summary, HLA-F expression, as shown in the above studies, can occur in both the cytoplasm and on the cell surface of EVT, in various configurations and at different stages of pregnancy.

#### 8.2.4. HLA-C

HLA-C is the only classical polymorphic HLA expressed on the placenta and has also been investigated in relation to pregnancy. Unlike the monomorphic non-classical HLAs, HLA-C is polymorphic, and as such, the paternal allele inherited by the fetus will differ from the mother’s [146]. HLA-C is reported to be expressed only in the placenta in the β2 m-associated form [147]. HLA-C is strongly expressed by EVT in the anchoring columns of the early first-trimester placenta (Figure 4) [148]. Hackmon et al. showed that HLA-C was strongly expressed on the cell surface of proliferating EVT cells in the cell column, in both villous sections and the EVT outgrowth model, but was internalized and localized to the cytoplasm as the EVT became invasive [100]. This early EVT expression of HLA-C is thought to be important in NK cell development, a process called “education” and “ licensing” of dNK, as a recent study has shown that trans presentation of IL-15 (a key proliferative factor for dNK) along with cognate HLA-C ligands results in reduced NK cell proliferation through engagement of either KIR2DL1 or KIR2DL2/3, indicating that inhibitory NK cell receptors contribute to dNK cell homeostasis and thus a healthy pregnancy [148,149]. They further showed that as gestation progressed and the placenta matured, HLA-C was also expressed by fetal mesenchymal and endothelial cells in the fetoplacental circulation. Quantitative methods demonstrated that HLA-C mRNA and protein levels were significantly higher in placentas from laboring deliveries than in those from elective cesarean sections. This finding may suggest a role for HLA-C in parturition [1].

## 9. The Maternal and the Fetal Intertalk at the Decidual–Placental Interface

The maternal–fetal intertalk refers to the interaction between the maternal uterine decidua and the fetal placental interface during implantation and placentation in a healthy pregnancy. The complex, multilevel, and beneficial interplay between the EVT and the maternal decidual white blood cells, macrophages, and T cells in this immune-tolerant process is affected by interactions between the non-classical HLA class I ligands expressed on the placenta and dNK, LILRB1/2, KIR, NKp, and T-cell immunoreceptors [75,102] as discussed in detail previously and summarized below.

### 9.1. HLA-G Receptors

**Figure 6:**
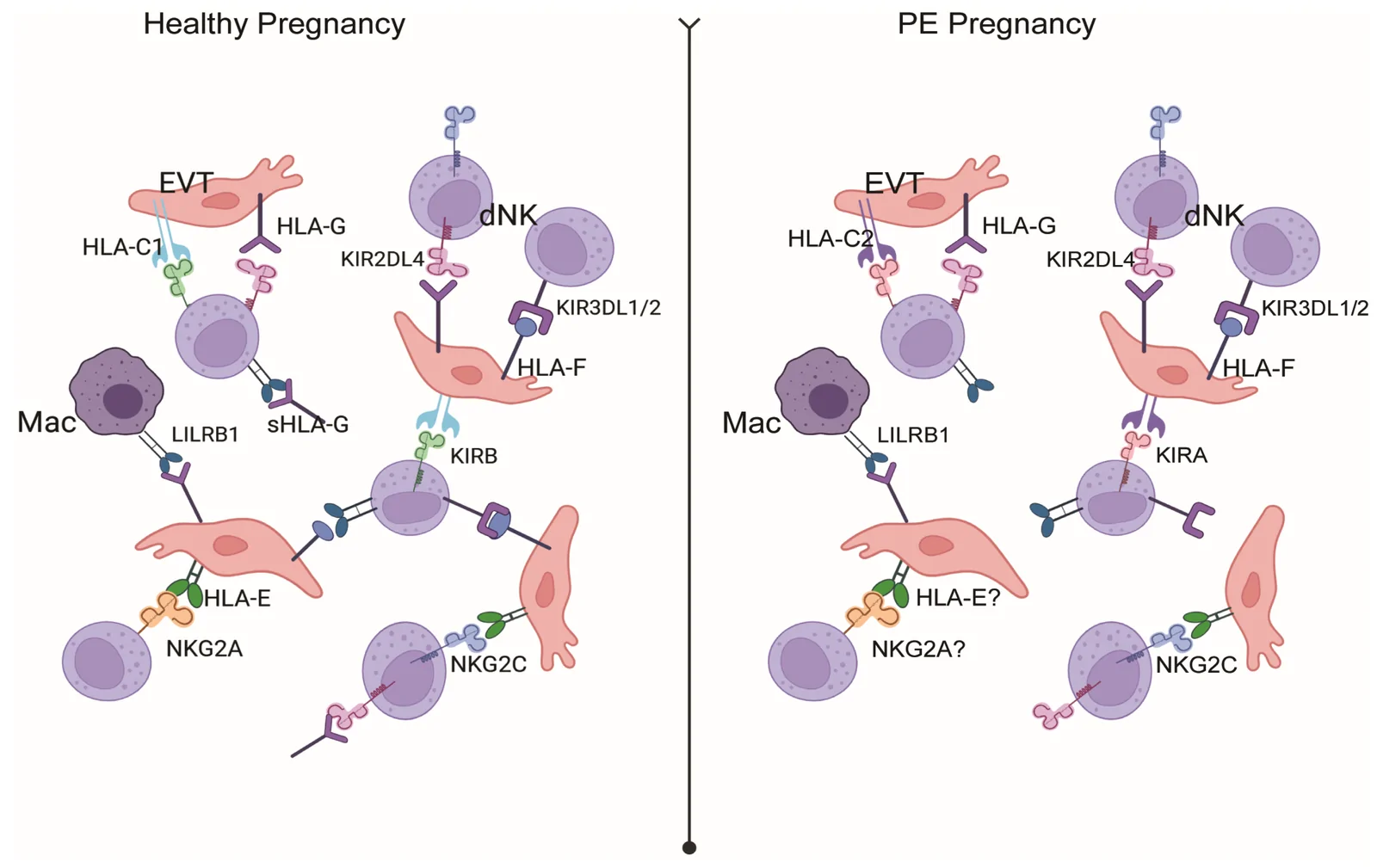
Maternal–fetal intertalk between HLA and dNK Receptors in Healthy and PE Pregnancies. In a healthy pregnancy, EVT (pink cells) express HLAG and release soluble HLA-G to interact with LILRB1 and KIR2DL4 receptors on dNK (purple cells); EVT express HLA-E, which binds NKG2A and NKG2C, while HLA-F interacts with KIR3DL1/2 receptors. HLAC1 and KIRB haplotypes predominate. In a PE pregnancy, HLA-G and sHLA-G levels are reduced. HLA-F levels are reduced, and HLA-C2 and KIRA haplotypes predominate. These changes alter the education and activity of dNK cells. Created in BioRender. Wright, T. (2026); https://BioRender.com/kz7kj0e (accessed on 7 April 2026).

Membrane-bound HLA-G binds to the KIR2DL4 activating receptor on dNK and to the inhibitory receptors LILRB1 and LILRB2 on macrophages [78]. LILRB1 shows a 3- to 4-fold preference for binding HLA-G [89,150]. The binding of both LILRB1 and KIR2DL4 by HLA-G homodimers has been reported to release IL-6, IL-8, and TNF-α [151] from decidual macrophages and dNK, respectively, resulting in a more robust response (Figure 6) [89,151].

All these receptors display immunoreceptor tyrosine-based inhibition motifs (ITIMs) in their cytoplasmic tails, where HLA-G exerts its inhibitory functions. Upon HLA-G binding, the ITIMs primarily recruit the protein tyrosine phosphatases SHP-1 and SHP-2 [150,152], which dephosphorylate key signaling pathways and inhibit cytotoxic effects on target cells [153].

On T cells, the CD8 co-receptor can also recognize and bind HLA-G, but it competes with LILRB1 for binding to HLA class I molecules; therefore, HLA-G can modulate the activation of cytotoxic T lymphocytes by blocking CD8 binding. The binding of HLA-G to its receptors on T cells inhibits the proliferation of these cells [154], the cytotoxic function of CD8+ T cells [155], and the alloreactivity of CD4+ T cells [156]. In addition, HLA-G has been reported to induce apoptosis of CD8+ cells [157,158]. This function regulates the balance between T helper (Th) 1 and Th2 cells, promoting polarization toward the Th2 subset. In fact, HLA-G decreases the production of IFNγ and tumor necrosis factor α (TNF-α) but increases the production of IL-3, IL-4, and IL-10 by Th2 [159].

In summary, a combined placental HLA-G interaction with inhibitory and activating receptors ultimately prevents the cytotoxic activation of dNK cells, decidual macrophages, and T cells and enhances their pro-angiogenic and pro-pregnancy effects. This data supports the hypothesis that HLA-G plays a crucial role in placentation and in suppressing the mother’s immune response against the placenta and fetus.

### 9.2. HLA-E Receptors

HLA-E binds to the CD94/NKG2A heterodimer inhibitory receptor on dNK cells with high affinity, suppressing cytotoxic activity and promoting maternal immune tolerance [113]. Additionally, HLA-E, like HLA-G, can interact with a variety of immunomodulatory receptors, including LILRB1 and LILRB2 on monocytes, and bind to NKG2C-activating receptors on dNK and T cells [82,160]. The molecular basis of the weaker binding to HLA-E of the CD94/NKG2C receptor is unclear; however, it is suggested that the change of the lysine for glutamic acid at the C-terminal, membrane-distal portion at residue 197 is important for altered affinity [91].

As mentioned previously, the HLA-E requires the supply of peptides from the classical HLA-A, -B, and -C, and the non-classical HLA-G. A peptide cleaved from the leader sequence of HLA-A,-B, or -C must bind to HLA-E for it to become a ligand for the conserved CD94/NKG2A receptor. There is dimorphism at the −21 position of the leader sequence supplied by HLA-B (−21 HLA-B), which encodes either threonine (T) or methionine (M) [91,161], thereby categorizing individuals into those who can provide functional peptides for high HLA-E expression and NKG2A ligation. NKG2A-driven “education” in MT or MM individuals results in NK cell populations that are phenotypically more diverse and exhibit increased functional potency [102,104]. The two alleles *A* and *G* of the single-nucleotide polymorphism (SNP) rs1050458 encode the two isoforms of the HLA-B leader peptide −21M and −21T, respectively [104]. A role for this *HLA-B* dimorphism is emerging in HIV control, immunotherapy in patients with leukemia, and graft-versus-host disease [162,163]. The exact maternal HLA→HLA-E→NKG2A pathway that affects pregnancy outcome remains unknown [164].

As noted previously, in the placenta, where classical HLA-A and B are not expressed, the non-classical HLA-G can enhance and stabilize the surface expression of HLA-E in target cells [112], which, through the CD94/NKG2A receptor, can exert inhibitory effects on NK cells. Furthermore, studies indicate that in multigravida women, the HLA-E/G complex has the highest affinity for the decidual activating receptors CD94/NKG2C and LILRB1, which promote the production of growth and angiogenic factors essential to normal placentation, compared to nulligravida women [10]. This supports our seminal theory of the crucial roles of HLA-G and HLA-E antigens in normal implantation and immunotolerance.

### 9.3. HLA-C Receptors

HLA-C antigen is the single classical polymorphic HLA expressed on the placenta, unlike the rest of the monomorphic non-classical placental HLAs. Consequently, the paternal allele inherited by the fetus may differ from the maternal allele [165].

The placental HLA-C antigen interacts with the NK-KIRs. As both HLA-C and KIR are highly polymorphic, every pregnancy is characterized by combinations of maternal KIR and fetal HLA-C variants. The maternal HLA-C genotype is also important to consider, as it may influence the repertoire and function of dNK cells through interactions between maternal KIR and her own HLA-C molecules during NK cell development, a process called “licensing” or “education” [166].

There are two KIR haplotypes, A and B. The KIR A haplotype only has inhibitory activity, while the KIR B haplotype can have additional excitatory activity. Quantitative RT-PCR and FACS studies of early decidual samples and matched blood have shown that both activating and inhibitory KIR2D specific for HLA-C are expressed at higher levels and on a greater proportion of dNK cells than in pbNK [167]. HLA-C alleles can also be divided into two groups, C1^+^HLA-C and C2^+^HLA-C, depending on the sequence of the KIR-binding site of the HLA molecules (KIR epitope).

KIR2DL2 and KIR2DL3 recognize HLA-C1, whereas KIR2DL1/S recognize HLA-C2. The frequency of NK cells positive for these KIRs is significantly higher in the decidua than in matched blood from the same patient [168].

It is thought that a balance between inhibitory and activating KIR receptor signaling, as observed in the KIR B haplotype, is another contributing factor for dNK licensing and results in a healthy pregnancy, while a strong inhibition of dNK activity, as is associated with the KIR A phenotype, may contribute to nonfunctional or cytotoxic dNK cells [169,170,171].

Prior seminal studies by Hiby et al. described the clinical significance of maternal KIR haplotype combinations and fetal group C alleles in pregnancy-related conditions, including the risk of implantation failure, miscarriage, PE, and low birth weight [170,172].

### 9.4. HLA-F Receptors

The placental function of HLA-F and its receptors is much less studied and understood. The unique open conformer state of HLA-F enables MHC Class I molecules to stabilize other receptors and other MHC Class I molecules [169,173]. HLA-F has been shown to assist in antigen presentation by binding to open conformers of MHC Class I, enabling a peptide-loading process that does not depend on TAP and tapasin. These HLA-F open conformers can interact with four different KIRs found on NK cells (see Figure 6). Among these are KIR3DL1 and KIR3DL2, which are inhibitory receptors due to their ITIMs, and the activating receptors KIR3DS1 and KIR2DS4 [97,143,174]. HLA-F-β2–microglobulin–peptide complex can also bind to the receptors LILRB1 and LILRB2 [160,175,176]. These receptor-mediated signals bias dNK toward a low-cytotoxic, pro-angiogenic phenotype, characterized by the secretion of GM-CSF and VEGF, which enhance extravillous trophoblast invasion and spiral artery remodeling [175].

The mechanisms underlying KIR receptor-HLA-F interactions at the maternal–fetal interface or periconceptionally remain poorly understood. To date, most studies of HLA-F and KIR cellular interactions have not focused specifically on pregnancy-related interactions. In a study of patients receiving IVF treatment, the abundance of dNK cells in the mid-secretory endometrium correlated with HLA-F expression, and dNK cell number and HLA-G expression were associated with achieving pregnancy in the subsequent IVF treatment cycle [177]. Future studies should integrate molecular, immunological, and clinical approaches to elucidate HLA-F’s mechanistic role in reproductive success [116].

## 10. The Immunology of the Maternal–Fetal Interface in Preeclampsia

As previously discussed, incomplete spiral artery remodeling causes malplacentation, which in turn results in malperfusion of the maternal–fetal interface during the prolonged first prodromal stage of PE. dNK cells have critical roles in regulating decidual vascular remodeling in conjunction with EVTs by producing a series of growth factors, cytokines, and chemokines. Staff et al. proposed that increased maternal systemic endothelial inflammation in PE leads to oxidative and inflammatory stress in the decidual compartment and inadequate remodeling of spiral arteries. This results in early-onset PE, often accompanied by FGR and a Type I phenotype [20,21].

To date, several studies have investigated dNK cell dysfunction in the initiation of PE. Some studies have reported that the number of dNK cells is significantly higher in PE than in normal pregnancies [178,179], whereas others have reported a significant decrease [179,180,181]. These discrepancies in results may stem from differences in sampling sites (i.e., decidua basalis, parietalis, or placental bed biopsy) or from the techniques and tests used (i.e., flow cytometry versus immunohistochemistry). Nonetheless, more valuable insight could be obtained from functional studies of dNK cells in PE.

Wallace et al. were the first to report that a reduction in the proportion of dNK cells in early pregnancy expressing KIR2DL/S1,3,5 and LILRB1 receptors was related to a high risk of subsequent preeclampsia [182]. Some studies have shown that the Natural cytotoxicity receptors expressed by NK (NKp44, NKp46, and NKp30) are also altered in PE. Indeed, a significantly reduced percentage of NKp46+ NK cells in the peripheral blood of women with PE, compared with women without PE, can be observed 3–4 months before the onset of PE [183]. Zhang et al. reported that preeclamptic decidual tissues retained many dNK cells with higher CD56 molecular density, indicating that a majority of dNK cells in PE are arrested in an immature state, with high expression of NKp46 and NKp3 [87]. These authors also showed that higher levels of TGF-β produced by decidual Treg cells downregulated IFN-γ, IL-8, and CD107a expression in NK cells and selectively modulated the proportions and function of specific dNK subsets in the preeclamptic decidua [87]. A recent single-cell sequencing study of decidual samples from 2 PE pregnancies showed a disproportionately high number of NK2 subtypes as compared with control samples. Differential gene expression analysis in NK cells identified decreased CCL5 expression and significant downregulation of ribosome-related genes in patients with PE. Interestingly, a variety of MHC-I molecules were also downregulated in PE patients compared with controls, with HLA-C showing the most significant reduction. Analysis of KEGG signaling enrichment also revealed significant alterations in signaling pathways related to NK cell ribosomal functions, allograft rejection, and Toll-like receptors [184]. In summary, growing evidence indicates that dNK cell function and phenotype are altered in PE pregnancies and may contribute to early pathogenic changes in the decidua [173].

Despite numerous studies, the etiology of PE still remains unknown. Yagel et al. hypothesize that incomplete spiral artery remodeling, systemic endothelial inflammation, and placental angiogenic imbalance all originate from maternal immunologic maladaptation, mediated by abnormal placental non-classical HLA expression, beginning as early as the initial stages of pregnancy and implantation. The next section will review recent relevant research, including this group’s original findings on dysregulation of non-classical HLA and HLA-C and their interactions with NK receptors at the maternal–fetal interface in PE (Figure 6).

### 10.1. HLA-G SNPs in PE

Increasing evidence suggests that dysregulation of HLA-G is strongly associated with PE. Several genetic polymorphisms in maternal HLA-G have been identified in women with PE. There are currently five known polymorphisms commonly found in all populations: one at codon 31 in exon 2; three in exon 3 at codons 93, 107, and 110; and a 14bp deletion in the 3′UTR of exon 8. Most of these are found at low frequency; the CAC-CAT polymorphism at codon 93 and the 14-bp deletion in the 3′-untranslated region (UTR) both occur at relatively high frequency [185]. Significant differences in allele, genotype, and haplotype distributions have been observed between normal and PE samples, suggesting an association between the HLA-G polymorphism and PE [186]. The relevance of the 14bp ins/del region in the 3′UTR has been suggested to impact the splicing and expression of HLAG isoforms. Allelic imbalance in heterozygous primary trophoblast samples manifested as differential expression of allele-specific mRNA transcripts involving a 14-bp insertion/deletion. The 14-bp del/del genotype correlated with notably reduced levels of G1 mRNA isoform and increased levels of G3 mRNA isoform. Additionally, this imbalance was linked to decreased HLA-G surface expression on primary trophoblast cells [187]. Indeed, Zhu et al. reported that in preeclamptic term placentas, both HLA-G mRNA and protein levels were significantly decreased compared with normal term placentas [188]. Hara et al. further reported attenuated expression of HLA-G protein on EVT in preeclamptic term placentas [189]. Another study reports low levels of HLAG mRNA and protein in the EVT of the placental bed in pregnancies with PE [190]. Interestingly, Dunk et al. have shown that in the most severe PE Type 1 cases with a growth-restricted infant, proximal and distal HLA-G are highly expressed in the EVT deep within the placental bed compared with healthy term controls [190]. Of note, both early severe PE cases, with or without FGR, were delivered at 28–34 wga and before term; thus, there may be a bias when compared to term placentas, as we know that expression decreases as pregnancy advances. Further research is needed to compare HLA-G expression levels in placentas of the same gestational age, characterize HLA-G isoforms, and precisely determine placental HLA-G expression levels in PE.

### 10.2. Soluble HLA-G in PE

Low levels of the soluble form of HLA-G (sHLA-G) in maternal plasma are strongly associated with both Type 1 and 2 PE [100,191,192,193]. Hackmon et al. were the first to report that maternal sHLA-G levels at term are lower in severe PE than in normal pregnancies. Subsequently, others reported lower levels in the earlier stages of pregnancy. A recent meta-analysis investigating the possible relationship between low sHLA-G levels in maternal blood at the beginning of pregnancy and subsequent PE included 5 studies and 668 subjects and found that low sHLA-G levels in early pregnancy are strongly positively correlated with subsequent PE [194]. Jacobsen et al. also report lower levels of sHLA-G in early and late-onset PE, however interestingly, in the postpartum period plasma they reported significantly higher sHLA-G levels in women who had had Type 1 PE compared to Type 2. sHLA-G was also elevated up to 3 years after early-onset Type 1 preeclampsia, suggesting an excessively activated immune system following this severe preeclampsia form, potentially contributing to future cardiovascular disease risk [192].

In summary, these studies increasingly support the downregulation of sHLA-G in PE as early as the first trimester. This downregulation may lead to a lack of maternal tolerance mediated by interactions among HLA-G, LILRB1, and KIR2DL4 with dNK cells and macrophages, as well as increased maternal inflammation observed in women with PE. Furthermore, they support the original theory that sHLA may serve as an early biomarker for PE screening.

### 10.3. HLA-E and HLA-B Dimorphism in PE

As mentioned earlier, HLA-E presents peptide leader sequences that influence its interactions with NKG2A on dNK. Recently, Moffett and Colucci demonstrated that genetic ablation of NKG2A in dams mated with wild-type males resulted in a suboptimal maternal vascular response during pregnancy, comparable to that observed in human PE. They also reported a genome-wide association study of 7219 pre-eclampsia cases, in which they found a 7% greater relative risk associated with the maternal *HLA-B* allele that does not favor NKG2A signaling. In addition, they showed that PE is less prevalent in women carrying the −21M HLA-B genotype, which favors NKG2A education, suggesting that the maternal HLA-B→HLA-E→NKG2A pathway optimizes pregnancy outcomes [102]. Interestingly, this education is linked to maternal, not fetal, MHC and is likely unrelated to pregnancy, as MHC-deficient dams produce more growth-restricted fetuses even when these fetuses express self-MHC [195].

In a recent study of placentas and decidual tissue from women with Type 1 PE, compared with preterm birth and healthy pregnancies, we found very weak HLA-E expression in the EVT of the third-trimester placental basal plate, with no differences between PE samples and controls. We observed strong HLA-E expression in the syncytiotrophoblast bilayer of the placenta; however, its role remains unknown. We also observed a significant reduction in HLA-E levels in endothelial cells of the placental vasculature in PE pregnancies compared with controls [1]. This endothelial expression of HLA-E is expected, and it is thought to play a regulatory role in coagulation, inflammation, and vascular homeostasis [196], which are all factors that PE compromises [197].

Hackmon et al. previously showed that HLA-E antigen expression on EVT was restricted to the very early gestation (Figure 5) [100]. This, together with the fact that the HLA-G/E complex has a very high affinity for the inhibitory NKG2A receptor, suggests that placental HLA-E and its complexes are essential antigens in normal pregnancies and in PE. It remains to be established if defects in their early expression and interaction contribute to the failure of maternal–fetal communication and the onset of PE.

### 10.4. HLA-F EVT Expression in PE

So far, studies on HLA-F polymorphisms linked to PE have not found any significant associations. A study examining specific HLA-F polymorphisms, including single-nucleotide polymorphisms (SNPs) rs1362126 (G/A), rs2523405 (T/G), and rs2523393 (A/G), along with their genotypes, haplotypes, and diplotypes in 192 controls and 164 PE cases, did not identify any connections with PE risk. However, a pattern suggesting the absence of certain HLA-F extended diplotypes in preeclampsia was noted [198].

HLA-F expression is increased on invading EVT in the first trimester, and growing evidence also links high HLA-F expression with invasive cancer progression and metastasis [199,200], further supporting a pro-invasive role of HLA-F. In a recent study by this group evaluating the placental bed in healthy and PE pregnancies, Dunk et al. found that EVT in PE samples exhibited significantly lower HLA-F expression, suggesting that this may reflect the reduced invasive capacity of these cells in PE [1]. This finding was validated by a single-cell sequencing study, which also reported lower mean fluorescent intensity for HLA-F in EVT cells from PE pregnancies [184,200]. This group has since demonstrated that HLA-F plays a role in trophoblast proliferation. Using overexpression and silencing methods in a trophoblast cell line, they showed that reduced HLA-F expression downregulated transcription and protein expression of Pyruvate Kinase Muscle isoform 2. Concurrently, the relative upregulation of lactylation at PKM2 K305 contributed to a decline in enzyme activity, further exacerbating glycolysis dysfunction [201]. Concentrations of pyruvate and lactate, but not glycogen or glucose, are reported to be significantly lower in placentas from mothers with severe preeclampsia, supporting evidence of inhibited glycolysis [202]. Collectively, these results suggest that HLA-F may play a significant role in metabolic abnormalities observed in the placentas of mothers with severe PE.

### 10.5. HLA-C and KIR Haplotypes in PE

Placental HLA-C, by interacting with the strong inhibitory KIR2DL1, may lead to low birth weight and increased PE risk, as this interaction suppresses activation rather than education of dNK cells [169,172,203]. *HLA-C* alleles can be divided into two groups, C1^+^HLA-C and C2^+^HLA-C, depending on the sequence of the KIR-binding site of the HLA molecules (KIR epitope). Interestingly, the possible combination of maternal NK KIR interactions with placental EVT HLA-C group was investigated by Hiby et al. [169,170], who reported that a mother with two *KIR A* haplotypes, when paired with a fetus carrying a group C2^+^ *HLA-C* allele, is associated with increased risk of PE. Interestingly, there is also a higher risk of implantation failure in IVF pregnancies, miscarriages, and low birth weight. There is a powerful inhibitory signal from a fetal C2^+^HLA-C when maternal NK cells lack activating KIR (not present on the *KIR A* haplotype). There is a protective effect of PE when a mother has an activating KIR for C2^+^ HLA-C on *KIR B* haplotypes.

Our group, on the other hand, investigated the frequency of the maternal KIR AA/fetal HLA-C2 combination in cases of severe PE and eclampsia and compared it to women with pregnancy-related hypertensive disorders. No association between HLA-C/KIR genetic variation and the risk of severe PE was found, leading to the proposed explanation that severe Type 1 PE represents a distinct pathological entity compared with mild PE [204]. In our study investigating the PE placental bed, HLA-C was highly expressed in the distal EVT compared to the proximal EVT. This was most pronounced in the severe Type 1 PE group compared with healthy pregnancies [1]. We suggest that the elevated expression of HLA-G and C in these severe PE cases may therefore reflect the undifferentiated status of the EVT in the PE placental bed and contribute to the defective utero-vascular transformation observed in Type 1 PE.

## 11. The Relationship Between Placental Antigen Expression and Soluble HLA Levels

Earlier studies from this group showed reduced levels of sHLA-G in amniotic fluid (AF) during normal term pregnancies compared with the second trimester [115]. These results were consistent with subsequent histological findings of a decrease in HLA-G EVT expression as pregnancy progresses [193]. Similarly, as discussed above, numerous studies have reported low levels of HLA-G in the placenta and EVT of PE pregnancies and low levels of sHLA-G in maternal serum in PE [115,193]. A few studies have found significantly low sHLA-G levels as early as the first trimester [191,192]. Collectively, these results suggest that placental expression of HLA-G mirrors the levels of sHLA-G observed in the maternal blood and have led us to postulate a direct correlation between distal EVT expression and soluble antigen levels across different maternal fluid compartments that will enable screening maternal blood for placental expression of non-classical HLAs early in pregnancy. Unfortunately, it is currently impossible to compare placental HLA-G with sHLA-G in cases of early-onset severe PE, as these pregnancies are delivered before term, and there are no adequate placentas that will serve as normal controls.

## 12. Future Research Avenues

The evidence described above supports the emerging potential roles of HLA-E, HLA-G, and the HLA-E/G complex, along with other placental HLAs, in immunosuppression and allograft acceptance during placentation and in PE (Figure 6). Furthermore, we hypothesize that early placental HLA antigen expression in PE can be detected in maternal serum as soluble forms, and that these soluble maternal serum levels may serve as accurate biomarkers and screening tools for PE.

In this context, our group is conducting research to test the hypothesis that placental HLA-G, HLA-E, and HLA-C, and their complexes, function as immunosuppressors during early placentation, thereby allowing normal placental development and acceptance. Based on prior evidence, we are convinced that further investigation in this field holds promise for elucidating the etiology of PE.

Further research is needed to clarify the essential role of maternal–fetal interactions in implantation in both healthy and PE pregnancies and to elucidate the complex mechanisms behind implantation and parturition. Additional research is also required to identify maternal soluble HLA-E and HLA-E/G complexes, compare soluble HLA forms in PE with those in normal pregnancies, and better define the functions of non-classical placental HLAs and their interactions with dNK cells during early placentation in both normal pregnancy and PE.

## 13. Conclusions

This review explores the fascinating field of immunology in pregnancy. This rapidly evolving area, marked by groundbreaking innovations, has the potential to transform our understanding of placentation, parturition, and PE, yet significant knowledge gaps remain. Importantly, accurate early screening for PE could enable early diagnosis and intervention, allowing testing of novel prevention strategies and treatments that could ultimately alter the clinical course and treatment of PE. This will undoubtedly improve maternal and fetal health outcomes and close the existing gap in current health disparities.

## Data Availability

No new data were created or analyzed in this study. Data sharing is not applicable to this article.
